# Prevalence of Methicillin-Resistant *Staphylococcus aureus* and Other *Staphylococcus* Species in Raw Meat Samples Intended for Human Consumption in Benin City, Nigeria: Implications for Public Health

**DOI:** 10.3390/ijerph13100949

**Published:** 2016-09-24

**Authors:** Etinosa O. Igbinosa, Abeni Beshiru, Lucy U. Akporehe, Faith E. Oviasogie, Owen O. Igbinosa

**Affiliations:** 1 SAMRC Microbial Water Quality Monitoring Centre, University of Fort Hare, Private Bag X1314, Alice 5700, South Africa; 2 Applied Microbial Process & Environmental Health Research Group, Department of Microbiology, Faculty of Life Sciences, University of Benin, Private Mail Bag 1154, Benin City 300001, Nigeria; bash_ab@rocketmail.com (A.B.); agimlucy7@gmail.com (L.U.A.); faith.oviasogie@uniben.edu (F.E.O.); 3 Department of Medicine, Obafemi Awolowo University Teaching Hospitals Complex, Ile-Ife 220005, Nigeria; oghosa2001@yahoo.com

**Keywords:** methicillin resistance, virulence gene, resistant gene, health risk, multi-drug resistant

## Abstract

The present study was designed to characterize methicillin-resistant staphylococci from raw meat. A total of 126 meat samples were obtained from open markets between February and April, 2015. Antimicrobial susceptibility testing was carried out using the disc diffusion method. Molecular profiling was conducted using 16S rRNA, *mec*A, *nuc*, and PVL gene signatures were detected by polymerase chain reaction assay. Fifty isolates of methicillin-resistant *Staphylococcus* spp. were detected in 26 (52%) pork, 14 (28%) beef and 10 (20%) chicken samples. The staphylococcal isolates were identified through partial 16S ribosomal ribonucleic acid (16S rRNA) nucleotide sequencing, and BLAST analysis of the gene sequence revealed 98%–100% staphylococcal similarity. All isolates from beef and chicken samples amplified the *mec*A gene, while 100% of the MRSA isolates amplified the PVL gene. The multidrug resistance profile (resistant to ≥1 antimicrobial agent in ≥3 classes of antimicrobial agents) of the staphylococcal isolates showed that 7 isolates were resistant to methicillin, penicillin, clindamycin, chloramphenicol, trimethoprim-sulfamethoxazole, kanamycin, amoxicillin, cloxacillin, erythromycin, vancomycin, and gentamycin. There was a significant regression effect from the multidrug-resistant profile on the number of isolates (*p* < 0.05) suggesting a consequence of the dissemination of resistant strains within bacterial populations. The findings of the present study indicate that raw meats in the Benin metropolis were possibly contaminated with pathogenic and multi-drug resistant staphylococci strains and therefore could constitute a risk to public health communities.

## 1. Introduction

Staphylococci are pathogens that can cause a range of diseases which are significant to public health [1] and are important food-borne pathogens [1,2]. They are considered the third most significant cause of disease in the world among reported food-borne illnesses [3] coupled with their pathogenicity, which depends on various bacterial surface components [4]. Though the precise role of a particular virulent determinant in relation to infection is difficult to establish, the production of leucocidal toxins, which are expressed by leucocidal genes detected in the genome of staphylococcal isolates recovered from infections resulting in deep and soft tissue infections (furunculosis, severe necrotizing pneumonia and cutaneous abscesses), suggests an important virulence factor in staphylococci: that they harbor the panton-valentine leucocidin (PVL) gene [1,5] which is an attribute for community-acquired methicillin-resistant staphylococci isolates [5]. The secretion of synergohymenotropic toxins resulting from the expression of the PVL gene forms pores in the membrane of host defense cells due to the synergistic action of two secretory proteins, which are designated as LukS-PV and LukF-PV. These secretory proteins (LukS-PV and LukF-PV) are encoded by two co-transcribed genes of a prophage integrated in the *S. aureus* chromosome [1,5]. The thermostable endonuclease gene (*nuc* gene) encodes the expression of the thermostable endonuclease enzyme, known to be an important pathogenic factor for *S. aureus.* The presence of such a gene in the genome of the staphylococcal cell increases the organism’s inherent capacity to initiate infection [1,6].

The use of antibiotics in humans and in animals (therapeutic, growth promotion and prophylactic) possibly led to the selective increase of resistance in bacterial populations [7]. In recent years, methicillin-resistant staphylococci have been identified in animal-derived food products worldwide [1,2,3,4,5,7,8,9,10,11,12,13,14]. The methicillin resistance of staphylococci is mediated by the *mec*A gene, which is carried by a mobile genetic element known as the staphylococcal cassette chromosome *mec* (SCC*mec*) [3]. The penicillin binding protein 2a (PBP2a) has a reduced affinity for beta-lactam antibiotics, resulting in resistance to most beta-lactam antimicrobial agents. Therefore, *mec*A or PBP2a are indispensable targets for methicillin-resistant staphylococci detection [6,13].

The detection of staphylococci in meat is often connected to poor hygienic practices during slaughtering, transportation, chopping, storage and points of sale by the individuals involved in the production process. Contaminated meat can transfer large amounts of staphylococci to stainless steel and polyethylene surfaces with other consumables in the same polythene packs and bags [12]. The emergence of antimicrobial-resistant pathogens has been associated with increased morbidity and mortality in patients and increased medical care costs, particularly in developing countries where there are limited, generally unaffordable therapeutic options [10]. Community-acquired methicillin-resistant staphylococcal infections and infections caused by methicillin-resistant staphylococci are of increasing concern and have been detected in frozen and ready-to-eat foods [9]. Foods contaminated with antibiotic-resistant bacteria represent a model medium for the transmission of antibiotic-resistant strains. This study was designed to detect and characterize methicillin-resistant staphylococci in raw meats from open markets in the Benin metropolis.

## 2. Materials and Methods

### 2.1. Collection of Samples

A total of 126 meat samples which included 40 beef samples, 56 pork samples and 30 chicken samples were obtained from Edaiken, New Benin, and the Oba market in Benin City, Edo State, Nigeria. The samples were transported to the laboratory in a cooler with ice for analysis within 2 h of collection.

### 2.2. Sample Preparation and Isolation

Samples were prepared by weighing and homogenizing 1 g of the sample and dissolving it in 9 mL of sterile distilled water. Thereafter, the sample was serially diluted. An aliquot of 100 µL of the 10^−1^, 10^−3^ and 10^−5^ dilution was transferred into a sterile Petri dish with the aid of Pasteur pipettes, and the sterilized molten media at 45 °C were poured into each labelled Petri dish. The plates were inverted and incubated at 37 °C under aerobic conditions for 24–48 h to allow bacterial growth as described by Cheesbrough [15]. After the incubation period, bacterial colonies on the culture plates were enumerated and expressed as colony-forming units. MRSA was isolated using 100 mL Mueller Hinton (MH) broth supplemented with NaCl (6.5%) and incubated at 37 °C for 20–24 h [16]. One millilitre (1.0 mL) of Mueller Hinton (MH) broth was added to Tryptic Soy Broth (TSB) and incubated overnight at 37 °C. Ten micro-litres (10 μL) of Tryptic Soy Broth (TSB) were spread on MRSA selective agar plate (CHROMagar^TM^ MRSA-ITK Diagnostics BV, Uithoorn, The Netherlands) and incubated 48 h at 37 °C. For 24 to 48 h, plates were observed and presumptive bacterial colonies were streaked and purified on Columbia agar plates with 5% sheep blood and incubated for 24 h at 37 °C. All isolates were confirmed to be *Staphylococcal aureus*, characteristically, by their typical colonial appearance (displaying golden-yellow colonies), a positive catalase test, and staining as Gram-positive cocci in clusters. Other staphylococci species were culturally and morphologically identified as coagulase-negative staphylococci (CNS) forming non-golden yellow and non-beta-hemolytic colonies [1].

### 2.3. Identification of the Staphylococcal Isolates

Staphylococcal colonies were identified by standard microbiological culture-based tests which included Gram-staining, catalase testing (using 3% hydrogen peroxide), indole, oxidase, coagulase, citrate, urease, Voges-prosakaser, DNase tests, sugar fermentation and the oxidation and fermentation of mannitol salt agar [17]. All tests were performed according to standard guidelines and *S. aureus* (ATCC 29213) was used as a positive control in each test protocol.

### 2.4. Molecular Identification by Polymerase Chain Reaction (PCR)

#### 2.4.1. Genomic DNA Extraction

Isolation of genomic DNA extraction was carried out as described by Igbinosa et al. [1]. Single colonies grown on Brain Heart Infusion agar were transferred to 1.5 mL of Brain Heart Infusion broth and cultures were grown on a shaker for 48 h at 28 °C. After this period, cultures were centrifuged at 4600 rpm for 5 min. The resulting pellets were re-suspended in 520 µL of TE buffer, 15 µL of 20% SDS and 3 µL of Proteinase K (20 mg·mL^−1^) were added. The mixture was incubated for 1 h at 37 °C, then 100 µL of 5 M NaCl and 80 µL of a 10% Cetyltrimethyl ammonium bromide (CTAB) solution in 0.7 M NaCl were added and mixed. The suspension was incubated for 10 min at 65 °C and kept on ice for 15 min. An equivalent amount of isoamyl alcohol:chloroform (1:24) was incorporated and afterward incubated on ice for 5 min and centrifuged at 7200 rpm for 20 min. The aqueous stage was conveyed to a fresh tube where isopropanol (1:0.6) was included and DNA precipitated at −20 °C for 16 h. DNA was recovered by centrifugation at 7200 rpm for 10 min, washed with 500 μL of 70% ethanol, air-dried ambient temperature for 3 h and liquefied in 50 μL of TE buffer. The extracted DNA was kept at −20 °C and thereafter used for genotypic detection and bacterial typing procedures.

#### 2.4.2. PCR for Identification of Staphylococcal Isolates

PCR identification of staphylococci was carried out as described by Ma et al. [18]. The PCR consisted of a final volume of 50 µL which included 8 µL DNA and 42 µL reaction consisting of 5× GoTaq green reaction, 10 mM of each dNTPs, 10 pmol each 27F: 5’-AGAGTTTGATCMTGGCTCAG-3’ and 1525R: 5’-AAGGAGGTGWTCCARCC-3’ specific for ~1500 bp conserved domain of the 16S rRNA gene of bacteria primers and 0.3 units of Taq DNA polymerase. PCR was carried out using the following thermal cyclic regime: an initial denaturation at 94 °C for 1 min, which was followed by 29 cycles of denaturation at 94 °C for 30 s, annealing at 50 °C for 1 min and an extension at 72 °C for 1.5 min, a final extension of 72 °C for 5 min and cooling to 4 °C. Electrophoresis of amplicons was performed with 1% agarose gel containing ethidium bromide (EtBr) 0.5 mg·L^−1^, for 1 h at 100 V in 0.5× TAE buffer (40 mM Tris-HCl, 20 mM Na-acetate, 1 mM EDTA, pH 8.5) and visualized under an UV transilluminator.

#### 2.4.3. Sequencing of the 16S rRNA Genes

Sequencing was carried out as described by Tamura et al. [19]. The purified DNA samples were sequenced with an Automated DNA sequencing Analyzer (ABI 3730X, ACGT Incorporation, Germantown, MD, USA) using 27F and 1525R primers. All DNA sequences were subjected to the method of Altschul et al. [20] for comparison with the Basic Local Alignment Search Tool (BLAST) program alignment tool from GenBank at the National Center for Biotechnology Information.

#### 2.4.4. Specific PCR for the Identification of *Staphylococcus Aureus*

The identity of *S. aureus* isolates were further confirmed using a species-specific primer that code for the thermonuclease (*nuc*) gene. This was achieved by adopting the method of Othman et al. [21]. The 25.0 μL reaction mixture contained 12.5 μL of PCR Master mix, 7.5 μL of PCR H_2_O, 1.0 μL of genomic DNA and 2.0 μL each of *nuc* primers (*nuc*-F: 5’-GCGATTGATGGTGATACGGTT-3’) and *nuc*-R: (5’-AGCCAAGCCTTGACGAACTAAAGC-3’) with amplicon size 280 bp. *S. aureus* (ATCC 29213) was used as a positive control while nuclease free water was used as a negative control. The PCR amplification protocol included a total of 37 cycles run under the following conditions: DNA denaturation at 94 °C for 60 s, primer annealing at 55 °C for 30 s, and DNA extension at 72 °C for 90 s. After the final cycle, at 72 °C for 3.5 min, and cooling to 4 °C. Electrophoresis of amplicons was performed with 1% agarose gel containing ethidium bromide (EtBr) 0.5 mg·L^−1^, for 1 h at 100 V in 0.5× TAE buffer (40 mM Tris-HCl, 20 mM Na-acetate, 1 mM EDTA, pH 8.5) and visualized under an UV transilluminator. 

#### 2.4.5. Specific PCR for the Identification of Methicillin-Resistant Staphylococcus Species

The detection of the *mec*A gene was carried out as described by Ahmed et al. [22]. The 25.0 μL volume of PCR reaction mixture contained 1.0 μL of genomic DNA, 12.5 μL of PCR Master mix, 7.5 μL PCR H_2_O and 2 μL each of *mec*A primers. *mec*A gene was amplified with the following primers: *mec*A-F: (5’-TCCAGATTACAACTTCACCAGG-3’); *mec*A-R: (5’-CCACTTCATATCTTGTAACG-3’) with amplicon size 162 bp. DNA amplification was carried out for 40 cycles according to the following protocol: denaturation at 94 °C for 30 s, annealing at 55 °C for 30 s, and extension at 72 °C for 1 min with a final extension at 72 °C for 5 min, and cooling to 4 °C. Electrophoresis of amplicons was performed with 1% agarose gel containing ethidium bromide (EtBr) 0.5 mg·L^−1^, for 1 h at 100 V in 0.5× TAE buffer (40 mM Tris-HCl, 20 mM Na-acetate, 1 mM EDTA, pH 8.5) and visualized under an UV transilluminator. 

#### 2.4.6. Detection Luk S/F Gene Sequence That Encodes Virulence Determinants in Staphylococci

This was conducted applying the protocol described by McDonald et al. [23]. The 25.0 μL volume of PCR reaction mixture contained 1.0 μL of genomic DNA, 2.0 μL each of *lukS*-PV and *lukF*-PVL primers, 12.5 μL of Red Taq Master mix and 7.5 μL PCR H_2_O. Primers *luk*-PV-1(5’-ACACA CTATGGCAATAGTTATTT-3’); and *luk*-PV-2(5’-AAAGCAATGCAATTGAT GTA-3’) with amplicon size 176 bp. Amplification was carried out for 40 cycles according to the following protocol: denaturation at 94 °C for 30 s, annealing at 55 °C for 30 s, and extension at 72 °C for 1 min with a final extension at 72 °C for 5 min, and cooling to 4 °C. Electrophoresis of amplicons was performed with 1% agarose gel containing ethidium bromide (EtBr) 0.5 mg·L^−1^, for 1 h at 100 V in 0.5× TAE buffer (40 mM Tris-HCl, 20 mM Na-acetate, 1 mM EDTA, pH 8.5) and visualized under an UV transilluminator.

### 2.5. Determination of the Antibiotic Resistance Profiles of Staphylococcal Isolates 

#### Antibiotic Susceptibility Testing

The staphylococcal isolates that were positively identified using the culture-based methods were subjected to antibiogram characterization. All the bacterial isolates were tested for resistance or sensitivity to different antibiotics using the standard disc diffusion method (Kirby Bauer test) [24]. For the disc diffusion assay, bacteria were grown between 18 and 24 h on Mueller-Hinton agar, harvested and then suspended in 0.85% sterile physiological saline solution adjusted to a 0.5 McFarland turbidity standard, corresponding to 10^8^ cfu·mL^−1^. The inoculum was streaked onto plates of Mueller-Hinton agar using a sterile cotton swab and impregnated with appropriate antibiotics. The antibiotics group, antimicrobial agents and disc content include Penicillins (Methicillin 5 µg, Cloxacillin 5 µg, Penicillin 10 µg, Amoxicillin 10 µg); Macrolides (Erythromycin 15 µg); Aminoglycosides (Gentamycin 10 µg, Kanamycin 30 µg); Lincosamides (Clindamycin 30 µg); Phenicols (Chloramphenicol 30 µg); Folates (Trimethoprim-Sulfamethoxazole 1.25 µg); and Glycopeptides (Vancomycin 30 µg) (Supplemental Material Table S1). The results were recorded after 24 h of incubation at 37 °C. Commercially available antibiotics discs, obtained from Mast Diagnostics, Merseyside, United Kingdom, were used to determine the resistance patterns of the isolates against 11 different antibiotics (1 dose/disc), grouped into 7 different classes of antibiotics. The diameter of the zone of inhibition around each disc was measured and interpreted as Resistant (R), Intermediate resistant (I) or Sensitive (S) in accordance with the recommended standard established by the Clinical Laboratory Standards Institute [25].

### 2.6. Statistical Analysis

All data were analyzed using SPSS statistics, version 21.0 (IBM^®^ Corporation, Armonk, New York, NY, USA). Antibiotic resistance and virulence factors of the meat isolates were compared using the analysis of variance (ANOVA) and Regression test. *p*-values of <0.05 were considered statistically significant.

## 3. Results

### 3.1. Distribution of the Staphylococci Isolates

The distributions of the staphylococcal isolates in the study are presented in Table 1. Statistical analysis reveals that there was no significant difference observed in the distribution of the staphylococcal isolates (*p* > 0.05). The PCR products of the 16S rRNA for the confirmation of the 50 isolates from the pork, beef and chicken samples and the partial sequence of the *Staphylococcus* species are shown in Supplemental Material Tables S2–S4, respectively.

### 3.2. Multidrug Resistance Pattern of the Staphylococcus Species

An interim standard definition for acquired resistance to antimicrobials as a method for proper description of multidrug-resistant (MDR) and extensively drug-resistant (XDR) profiles of bacterial isolates of public health concern have been described by a group of international experts. These international experts are part of a joint initiative by the European Centre for Disease Prevention and Control (ECDC), and the Centre for Disease Prevention and Control (CDC). In their conclusion, non-susceptible to ≥1 agent in ≥3 antimicrobial categories were considered MDR, while non-susceptible to ≥1 agent in ≥2 antimicrobial categories were considered XDR [26]. This was used as the standard for the characterization of MDR profile of staphylococcal isolates in this study. 

Overall, 50 isolates from this study were found to be resistant to multiple drugs tested. The details of the multidrug-resistant *S. aureus* are listed in Table 2.

### 3.3. Detection of Resistance and Virulence Genes from the Meat Samples

**Figure 1:**
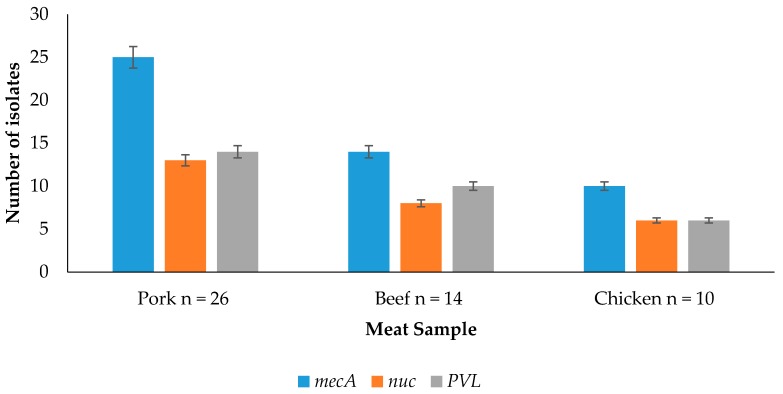
Detection of resistance and virulence genes obtained from pork, beef and chicken.

Detection of resistance and virulence genes obtained from pork, beef and chicken is shown in Figure 1. It shows that for pork isolates, 25/26 (96.15%) of the isolates amplified the *mec*A gene, 13/26 (50%) amplified the *nuc* gene, and 14/26 (53.85%) amplified the PVL gene. For beef isolates, 14/14 (100%) of the isolates amplified the *mec*A gene, 8/14 (57.14%) amplified the *nuc* gene and 10/14 (71.43%) amplified the PVL gene. For the chicken isolates, 10/10 (100%) of the isolates amplified the *mec*A gene, 6/10 (60%) amplified the *nuc* gene, and 6/10 (60%) amplified the PVL gene.

### 3.4. Detection of Resistance and Virulence Genes from the Staphylococcus Species

**Figure 2:**
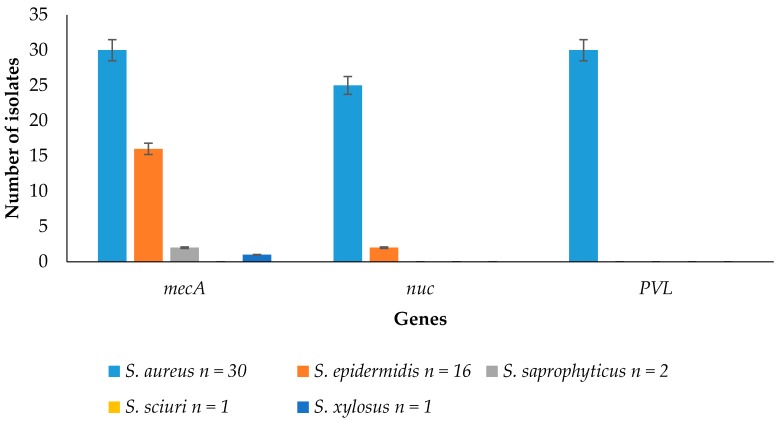
Detection of virulence and resistance genes from *Staphylococcus* spp.

Resistance and virulence genes detected from the *Staphylococcus* species are shown in Figure 2. It shows that 30/30 (100%) of *Staphylococcus aureus*; 16/16 (100%) of *Staphylococcus epidermidis*; 2/2 (100%) of *Staphylococcus saprophyticus*; and 1/1 (100%) of *Staphylococcus xylosus* amplified the *mec*A gene. Moreover, 25/30 (83.33%) of *Staphylococcus aureus* and 2/16 (12.5%) of *Staphylococcus epidermidis* amplified the *nuc* gene. In addition, 30/30 (100%) of *Staphylococcus aureus* amplified the PVL gene.

## 4. Discussion

### 4.1. Distribution of the Staphylococci Isolates

Distributions of the staphylococcal isolates from pork, beef and chicken samples in an open market in Benin City have been determined. Information from the study reveals significant amount of staphylococci detected in pork, beef and chicken samples investigated. These suggest raw meat samples from open markets in Benin metropolis are handled by human which could be a potential carrier of staphylococci isolates. An investigation by Diederen et al. [8] in the Netherlands revealed that 2.5% of the meat samples (pork and beef) harbored methicillin-resistant *Staphylococcus aureus* isolates. This is significantly lower compared to the findings in this study, which could be a result of differences in sampling sites, as Diederen et al. [8] obtained 83.87% in meat samples from controlled environments (supermarkets and grocery shops) while the meat samples investigated in this study were obtained from open markets. Vanegas-Lopez et al. [4] revealed that in their study, 149/149 staphylococci isolates were resistant to oxacillin, which shares the same antimicrobial group with methicillin from Columbian foods. This finding is significantly higher compared to the findings in this study, which could be attributed to other food sources such as milk cream and fruit salad which were not investigated in this study. Yang et al. [27] also revealed that of the 550 ready-to-eat food samples investigated in China, 69 (12.5%) were positive for *S. aureus*. This significantly is lower than the finding in the present study, 30 (60%). Staphylococci detected by Kumar et al. [28] revealed 13 (68.42%) from fresh seafood in North Mumbai, India, compared to the 50 found in this study (39.68%). However, Kumar et al. [28] also reported that 3 (15.78%) were *S. aureus* which was significantly lower than our finding of 30 (60%). Contreras et al. [29] also revealed that MRSA was detected in 32% of the hamburgers and 8% of the sandwiches investigated in Brazil, which was within the findings of our study.

### 4.2. Multidrug Resistance Pattern of the Staphylococcus Species

There was a significant regression (*r* (multiple R) = 0.7707) of the multidrug-resistant profile on the number of isolates (*p* < 0.05). This could be a consequence of the continuous incorporation of antimicrobials as growth promoters into the feeds of the animals, or the extensive use of these antimicrobials as prophylactic and therapeutic agents. Furthermore, human activities from the abattoir environment down to the open markets where they are available for sale to consumers could also be a predisposing factor, as *Staphylococcus aureus* have been reported by several authors to be a resident of the human nostril. Yang et al. [27] revealed that 75.8% (47/62) of the methicillin-susceptible *S. aureus* isolates and all of the MRSA 100% (7/7) isolates were resistant to three or more antibiotics. Similar study by Alexandra et al. [30] showed marked resistance to the action of beta-lactams (100%), lincosamides and macrolides (clindamycin and erythromycin 80%), aminoglycosides (gentamycin and kanamycin 20%), and folate synthesis inhibitors (sulfamethoxazole/trimethoprim 42%). These findings were in accordance with the findings in the present study, as all isolates were resistant to the action of beta-lactams (penicillins 100%), lincosamides (clindamycin 98%), macrolides (erythromycin 88%), aminoglycosides (gentamycin 30%, kanamycin 100%), and folate synthesis inhibitor (trimethoprim/sulfamethoxazole 100%). This significantly increases the pathogenic properties of staphylococci, making it an important public health issue due to its ability to cause nosocomial infection as well as community-associated infections. Furthermore, these infections are usually treated with beta-lactam antibiotics and staphylococci have developed marked resistance to these antibiotics. Contamination of raw food products by staphylococci should be monitored to identify probable threats of methicillin-resistant staphylococci infections for consumers [1]. 

### 4.3. Detection of Resistance and Virulence Genes from the Meat Samples

The presence of the *mec*A gene in staphylococci codes for methicillin resistance. It has been reported by Igbinosa et al. [1] that the detection of the *mec*A gene in staphylococci is a gold standard for the detection of methicillin-resistant staphylococci, as it codes for the presence of the penicillin binding protein 2a (PBP2a), which has a low affinity for beta-lactam antimicrobials. In the present study, 98% of the isolates that were resistant to methicillin using culture based method prove to harbor the *mec*A gene thus genetically resistant to the action methicillin. It was observed that there was no significant difference in the respective genes detected from the meat sources (*p* > 0.05). This could result to the fact that all samples were from animal sources and reports have revealed that they are usually treated with antimicrobials. Dissemination of antimicrobials resistant and virulent genes within bacterial population could be another important factor. Vanegas-Lopez et al. [3] showed that all 149/149 isolates that showed resistance to oxacillin using the conventional method amplified the *mec*A gene. Yang et al. [27] revealed that 6/7 (85.71%) that were methicillin-resistant conventionally harbored the *mec*A gene. The *mec*A gene was detected by Kumar et al. [28] by PCR in 10/13 (76.92%) methicillin-resistant coagulase-negative staphylococci (MR-CoNS) and 1/3 (33.33%) of the MRSA strain, respectively. This does not correspond to the findings in the present study. The *nuc* gene which expresses the thermostable endonuclease enzyme is specific to *Staphylococcus aureus.* However, these same genes were also detected in 2/16 (12.5%) *Staphylococcus epidermidis* in the present study which could be a result of horizontal gene transfer within the staphylococci isolates, which calls for further investigation. The panton-valentine leucocidin (PVL) gene is a virulent determinant which makes the staphylococci more virulent. It is found mainly among community-associated staphylococcal isolates. It has also been reported that methicillin-resistant *Staphylococcus aureus* strains are able to synthesize different kinds of enterotoxins alone or in association, providing further evidence that methicillin-resistant *Staphylococcus aureus* may also be involved in food poisoning outbreaks [14].

### 4.4. Detection of Resistance and Virulence Genes from the Staphylococcus Species

Importantly, MRSA-contaminated meat can serve as vehicle for the dissemination of antibiotic-resistant bacteria associated with risks to human health. Also, the occurrence of methicillin-resistant staphylococci from meats in open markets confirms that methicillin-resistant staphylococci are no longer only a problem for hospitals because they have entered the food chain, suggesting the dissemination of these resistant traits through horizontal gene transfer [1,2,3,4,5,6,7,8,9,10,12]. The tendency of *Staphylococcus epidermidis* to harbor the *nuc* gene could be attributed to the transfer of mobile genetic elements or horizontal gene transfer within bacterial populations as these isolates. A statistically significant difference was observed in the detection of the respective genes from the isolates (*p* < 0.05). Information from this study revealed that raw meat sold in Benin City had low bacteriological quality, raising concerns about its potential for the transmission of foodborne infections. Previous food safety research focused more on methicillin-resistant *Staphylococcus aureus.* However, this study evaluates the presence of both methicillin resistant *Staphylococcus aureus* and coagulase negative *Staphylococcus* spp. in meats. More research is needed to quantify the risk of colonization of consumers with methicillin resistant staphylococci via the food chain and to monitor a possible evolution of isolates with a potentially higher pathogenic attribute, i.e., staphylococcal enterotoxin gene content. More so, *mec*C has also been reported as an indispensable target for staphylococci in food animals and as such should be watched for together with the *mec*A for resistance to methicillin in further studies. The findings of this study highlight the occurrence of *Staphylococcus* spp. in raw meat sold in open markets in the Benin metropolis. Continuous monitoring of this pathogen is important for the surveillance, prevention and control of its associated diseases.

## 5. Conclusions

The presence and antimicrobial susceptibility profile of methicillin-resistant staphylococci from pork, beef and chicken have been characterized using standard culture-based methods and polymerase chain reaction assays. The incidence of methicillin-resistant staphylococci from retail raw meat confirms methicillin-resistant staphylococci is no longer only a problem for hospitals and has also entered the food chain, suggesting gene transfer of bacterial pathogens. The findings of this study indicate that raw meat in the Benin metropolis was contaminated with pathogenic and multidrug-resistant staphylococci strains. This highlights the need to implement suitable and appropriate control strategies to reduce complications and prevent the dissemination of resistant and virulent staphylococci isolates in the environment.

## Figures and Tables

**Table 1:** Distribution of staphylococcal isolates obtained from pork, beef and chicken carcasses.

*Staphylococcus* Species	Pork Isolates *n* = 26 (%)	Beef Isolates *n* = 14 (%)	Chicken Isolates *n* = 10 (%)	Total *n* = 50 (%)	*p*-Value
*S. aureus*	14 (53.85)	10 (71.43)	6 (60)	30 (60)	0.868
*S. epidermidis*	9 (34.62)	4 (28.57)	3 (30)	16 (32)	0.756
*S. saprophyticus*	2 (7.69)	-	-	2 (4)	0.422
*S. sciuri*	1 (3.85)	-	-	1 (2)	0.421
*S. xylosus*	-	-	1 (10)	1 (2)	0.421
Total	26 (100)	14 (100)	10 (100)	50 (100)	

**Table 2:** Multidrug-resistant *Staphylococcus* spp. isolated from pork, beef and chicken carcasses.

Phenotype Resistance	Number of Isolates *n* = 50
MET^R^, PEN^R^, CLN^R^	50
MET^R^, PEN^R^, CLN^R^, CHL^R^, SXT^R^, KAN^R^	50
MET^R^, PEN^R^, CLN^R^, CHL^R^, SXT^R^, KAN^R^, AMX^R^	48
MET^R^, PEN^R^, CLN^R^, CHL^R^, SXT^R^, KAN^R^, AMX^R^, CLX^R^	47
MET^R^, PEN^R^, CLN^R^, CHL^R^, SXT^R^, KAN^R^, AMX^R^, CLX^R^, ERY^R^	41
MET^R^, PEN^R^, CLN^R^, CHL^R^, SXT^R^, KAN^R^, AMX^R^, CLX^R^, ERY^R^, VAN^R^	29
MET^R^, PEN^R^, CLN^R^, CHL^R^, SXT^R^, KAN^R^, AMX^R^, CLX^R^, ERY^R^, VAN^R^, GEN^R^	7

**Legend:** MET: Methicillin; PEN: Penicillin; AMX: Amoxicillin; CLX: Cloxacillin; ERY: Erythromycin; GEN: Gentamycin; CLN: Clindamycin; CHL: Chloramphenicol; SXT: Trimethoprim-Sulfamethaxazole; VAN: Vancomycin; KAN: Kanamycin. ^R^: Resistant.

## Supplementary Materials

The following are available online at www.mdpi.com/1660-4601/13/10/949/s1. Table S1: Antimicrobial agents, disc content and zone diameter interpretative standards for *Staphylococcus* species, Table S2: Staphylococci species isolated from pork samples, Table S3: Staphylococci species isolated from beef samples, Table S4: Staphylococci species isolated from chicken samples.

## References

[1] Igbinosa E.O., Beshiru A., Akporehe L.U., Ogofure A.G. (2016). Detection of methicillin-resistant staphylococci isolated from food producing animals: A public health implication. Vet. Sci..

[2] Wang X., Tao X., Xia X., Yang B., Xi M., Meng J., Zhang J., Xu B. (2013). *Staphylococcus aureus* and methicillin-resistant *Staphylococcus aureus* in retail raw chicken in China. Food Control.

[3] Vanegas-López M.C., Moreno E.J., Rueda R.V., Chirivi S.J., Garzón A., Arévalo A.S., Martínez F.M., Gardeazábal P.A., Baquero C. (2012). Methicillin-resistant *Staphylococcus aureus* (MRSA) isolated from Colombian foods. Can. Cent. Acad. Art Sci..

[4] Khalifa S.M., Abdel-Rhman H.S.H., Abd-El-Galil K.H., Habib E., Barwa R. (2014). Occurrence and characterization of *Staphylococcus aureus* in meat and meat products marketed in Mansoura, Egypt. Egypt. J. Med. Microbiol..

[5] Beshiru A., Igbinosa I., Igbinosa E. (2016). Antimicrobial resistance of methicillin-resistant staphylococci isolated from food producing animal. 17th International Congress on Infectious Diseases. Int. J. Infect. Dis..

[6] Mirzaei H., Farhoudi H., Tavassoli H., Farajli M., Monadi A. (2012). Presence and antimicrobial susceptibility of methicillin resistant *Staphylococcus aureus* in raw and pasteurized milk and ice cream in Tabriz by culture and PCR techniques. Afr. J. Microbiol. Res..

[7] Suleiman A., Zaria L.T., Grema H.A., Ahmadu P. (2013). Antimicrobial resistant coagulase positive *Staphylococcus aureus* from chickens in Maiduguri, Nigeria. Sokoto J. Vet. Sci..

[8] Diederen B.M.W., van Lo I.H.M., Savelkoul P., Woudenberg J.H.C., Roosendaal R., Verhulst C., van Keulen P.H.J., Kluytmans J.A.J.W. (2007). Low prevalence of non-typable methicillin-resistant *Staphylococcus aureus* in meat products in the Netherlands. Session 6: Antimicrobial Resistance.

[9] De Boer E., Zwartkruis-Nahuis J.T.M., Wit B., Huijsdens X.W., de Neeling A.J., Bosch T., van Oosterom R.A.A., Vila A., Heuvelink A.E. (2009). Prevalence of methicillin-resistant *Staphylococcus aureus* in meat. Int. J. Food Microbiol..

[10] Otalu O., Junaidu K., Chukwudi E.O., Jarlath V.U. (2011). Multi-drug resistant coagulase positive *Staphylococcus aureus* from live and slaughtered chickens in Zaria, Nigeria. Int. J. Poult. Sci..

[11] Youssef A.I., Hamed D.M. (2012). Methicillin resistant *Staphylococcus aureus* (MRSA) associated with arthritis in broiler farms in Ismailia province, Egypt and its zoonotic potential significance. Suez Canal Vet. Med. J..

[12] Karmi M. (2013). Prevalence of methicillin-resistant *Staphylococcus aureus* in poultry meat in Qena, *Egypt*. Vet. World.

[13] Yamada K., Wanchun J., Ohkura T., Murai A., Hayakawa R., Kinoshita K., Mizutani M., Okamoto A., Namikawa T., Ohta M. (2013). Detection of methicillin-resistant *Staphylococcus aureus* using a specific anti-PBP2a chicken IgY antibody. Jpn. J. Infect. Dis..

[14] Nnachi U.A., Emele E.F., Ukaegbu O.C., Agah V.M., Udu-Ibiam E.O., Chukwu O.S., Agwu M.M. (2014). Prevalence of methicillin-resistant *Staphylococcus aureus* (MRSA) in raw meat and meat handlers in Onitsha, Nigeria. Eur. J. Prev. Med..

[15] Cheesbrough M. (2000). Microbiological test. District Laboratory Practice in Tropical Countries.

[16] Rinsky J.L., Nadimpalli M., Wing S., Hall D., Baron D., Price L.B., Larsen J., Stegger M., Stewart J., Heaney C.D. (2013). Livestock-associated methicillin and multidrug-resistant *Staphylococcus aureus* is present among industrial, not antibiotic-free livestock operation workers in North Carolina. PLoS ONE.

[17] Cruickshank R., Duguid J.P., Marmion B.R., Swain R.H. (1975). Medical Microbiology.

[18] Ma A., Lv D., Zhuang X., Zhuang G. (2013). Quorum quenching in culturable phyllosphere bacteria from tobacco. Int. J. Mol. Sci..

[19] Tamura K., Dudley J., Nel M., Kumar S. (2007). MEGA 4: Molecular evolutionary genetics analysis (MEGA) software version 4.0. Mol. Biol. Evol..

[20] Altschul S.F., Madden T.L., Schaffer A.A., Zhang J., Zhang Z., Miller W., Lipman D.J. (1997). Gapped BLAST and PSIBLAST: A new generation of protein database search programs. Nucleic Acids Res..

[21] Othman E.H., Merza S.N., Jubrael S.M. (2014). Nucleotide sequence analysis of methicillin-resistant *Staphylococcus aureus* in Kurtdistan Region-Iraq. J. Univ. Zakho.

[22] Ahmed B.O., Elmekki A.M., Omer E.E., Elhassan M. (2014). Molecular detection of methicillin-resistant *Staphylococcus aureus* in patients with urinary tract infections in Khartoum State. J. Sci. Technol..

[23] McDonald R.R., Antonishyn A.N., Hansen T., Snook A.L., Nagle E., Mulvey R.M., Levett N.P., Horsman B.G. (2005). Development of a triplex real-time PCR assay for detection of panton-valentine leucocidin toxin genes in clinical isolates of methicillin-resistant *Staphylococcus aureus*. J. Clin. Microbiol..

[24] Bauer A.W., Kirby M.M.W., Sherris J.C., Turck M. (1966). Antibiotic’s susceptibility testing by a standardized single disk method. Am. J. Clin. Pathol..

[25] Clinical and Laboratory Standards Institute (CLSI) (2014). Performance Standards for Antimicrobial Susceptibility Testing.

[26] Magiorakos A.P., Srinivasan A., Carey R.B., Carmeli Y., Falagas M.E., Giske C.G., Harbarth S., Hindler J.F., Kahlmeter G., Olsson-Liljequist B. (2012). Multidrug-resistant, extensively drug-resistant and pandrug-resistant bacteria: An international expert proposal for interim standard definitions for acquired resistance. Clin. Microbiol. Infect..

[27] Yang X., Zhang J., Yu S., Wu Q., Guo W., Huang J., Cai S. (2016). Prevalence of *Staphylococcus aureus* and methicillin-resistant *Staphylococcus aureus* in retail ready-to-eat foods in China. Front. Microbiol..

[28] Kumar R.G.L., Kasim K.A., Lekshmi M., Nayak B.B., Kumar S. (2016). Incidence of methicillin-resistant staphylococci in fresh seafood. Adv. Microbiol..

[29] Contreras A.P.C., da Silva N.N.S., Ferreira G.C.D., Ferreira D.J., Almeida D.C.R. (2015). Prevalence of methicillin-resistant *Staphylococcus aureus* in raw hamburgers and ready-to-eat sandwiches commercialized in supermarkets and fast food outlets in Brazil. Food Nutr. Sci..

[30] Alexandra F., Britta K., Gladys K., Beatriz G., Katja A., Jens-André H., Annemarie K., Juliane B., Bernd A., Bernd-Alois T. (2011). Methicillin susceptible and resistant *Staphylococcus aureus* from farm to fork impact on food safety. Tehnol. Mesa.

