# Supplementary material for: Prevalence of Methicillin-Resistant Staphylococcus aureus and Other Staphylococcus Species in Raw Meat Samples Intended for Human Consumption in Benin City, Nigeria: Implications for Public Health
Source: Int J Environ Res Public Health. 2016 Sep 24;13(10):949. doi: 10.3390/ijerph13100949 (PMC5086688; doi:10.3390/ijerph13100949)
Supplement: Supplementary file 1 [file ijerph-13-00949-s001.pdf]

Supplementary Materials: Prevalence of Methicillin-Resistant *Staphylococcus aureus* and Other *Staphylococcus* Species in Raw Meat Samples Intended for Human Consumption in Benin City, Nigeria: Implications for Public Health

Etinosa O. Igbinosa, Abeni Beshiru, Lucy U. Akporehe, Faith E. Oviasogie and Owen O. Igbinosa

Table S1. Antimicrobial agents, disc content and zone diameter interpretative standards for *Staphylococcus* species.

Antibiotics Group	Antimicrobial Agent	Disc Content	Zone Diameter (mm)		
			Resistant	Intermediate	Sensitive
Penicillins	Methicillin	5 µg	≤9	10–13	≥14
	Cloxacillin	5 µg	≤10	11–12	≥13
	Penicillin	10 µg	≤28	-	≥29
	Amoxicillin	10 µg	≤19	-	≥20
Macrolides	Erythromycin	15 µg	≤13	14–22	≥23
Aminoglycosides	Gentamycin	10 µg	≤12	13–14	≥15
	Kanamycin	30 µg	≤13	14–17	≥18
Lincosamides	Clindamycin	2 µg	≤14	15–20	≥21
Phenicols	Chloramphenicol	30 µg	≤12	13–17	≥18
Folates	Trimethoprim-Sulfamethoxazole	1.25 µg	≤10	11–15	≥16
Glycopeptides	Vancomycin	30 µg	-	-	≥15

Source: CLSI [1].

Table S2. Staphylococci species isolated from pork samples.

Isolates Code	Identification	Max Score	Total score	Query Cover %	E-Value	Identity %	Accession
P1	*Staphylococcus aureus*	97.6	97.6	100%	1e-17	100%	FN646074.1
P2	*Staphylococcus aureus*	85.7	85.7	87%	5e-14	100%	JF170962.1
P3	*Staphylococcus aureus*	85.7	85.7	87%	5e-14	100%	JF170962.1
P4	*Staphylococcus aureus*	85.7	85.7	87%	5e-14	100%	JF170962.1
P5	*Staphylococcus aureus*	97.6	97.6	100%	1e-17	100%	FN646074.1
P6	*Staphylococcus aureus*	97.6	97.6	100%	1e-17	100%	FN646074.1
P7	*Staphylococcus aureus*	85.7	85.7	87%	5e-14	100%	JF170962.1
P8	*Staphylococcus aureus*	85.7	85.7	87%	5e-14	100%	JF170962.1
P9	*Staphylococcus aureus*	85.7	85.7	87%	5e-14	100%	JF170962.1
P10	*Staphylococcus aureus*	97.6	97.6	100%	1e-17	100%	FN646074.1
P11	*Staphylococcus aureus*	85.7	85.7	87%	5e-14	100%	JF170962.1
P12	*Staphylococcus aureus*	85.7	85.7	87%	5e-14	100%	JF170962.1
P13	*Staphylococcus aureus*	97.6	97.6	100%	1e-17	100%	FN646074.1
P14	*Staphylococcus aureus*	85.7	85.7	87%	5e-14	100%	JF170962.1
P15	*Staphylococcus epidermidis*	89.7	89.7	100%	3e-15	98%	CP010942.1
P16	*Staphylococcus epidermidis*	89.7	89.7	100%	3e-15	98%	CP000029.1
P17	*Staphylococcus epidermidis*	89.7	89.7	100%	3e-15	98%	CP009046.1
P18	*Staphylococcus epidermidis*	89.7	89.7	100%	3e-15	98%	CP010942.1
P19	*Staphylococcus saprophyticus*	97.6	97.6	100%	1e-17	100%	KT372842.1
P20	*Staphylococcus epidermidis*	89.7	89.7	100%	3e-15	98%	CP000029.1
P21	*Staphylococcus epidermidis*	89.7	89.7	100%	3e-15	98%	HG813242.1
P22	*Staphylococcus sciuri*	95.6	95.6	100%	5e-17	100%	FN646076.1
P23	*Staphylococcus epidermidis*	89.7	89.7	100%	3e-15	98%	HG326660.1
P24	*Staphylococcus epidermidis*	89.7	89.7	100%	3e-15	98%	CP009046.1
P25	*Staphylococcus saprophyticus*	97.6	97.6	100%	1e-17	100%	KT372841.1
P26	*Staphylococcus epidermidis*	89.7	89.7	100%	3e-15	98%	AF269843.1

Table S3. Staphylococci species isolated from beef samples.

Isolates Code	Identification	Max Score	Total Score	Query Cover %	E-Value	Identity %	Accession
B1	*Staphylococcus epidermidis*	89.7	89.7	100%	3e-15	98%	FJ577582.1
B2	*Staphylococcus aureus*	97.6	97.6	100%	1e-17	100%	FN646074.1
B3	*Staphylococcus aureus*	85.7	85.7	87%	5e-14	100%	JF170962.1
B4	*Staphylococcus aureus*	97.6	97.6	100%	1e-17	100%	FN646074.1
B5	*Staphylococcus epidermidis*	89.7	89.7	100%	3e-15	98%	FJ577582.1
B6	*Staphylococcus aureus*	85.7	85.7	87%	5e-14	100%	JF170962.1
B7	*Staphylococcus epidermidis*	89.7	89.7	100%	3e-15	98%	CP000029.1
B8	*Staphylococcus aureus*	97.6	97.6	100%	1e-17	100%	FN646074.1
B9	*Staphylococcus aureus*	85.7	85.7	87%	5e-14	100%	JF170962.1
B10	*Staphylococcus aureus*	85.7	85.7	87%	5e-14	100%	JF170962.1
B11	*Staphylococcus aureus*	97.6	97.6	100%	1e-17	100%	FN646074.1
B12	*Staphylococcus aureus*	85.7	85.7	87%	5e-14	100%	JF170962.1
B13	*Staphylococcus aureus*	97.6	97.6	100%	1e-17	100%	FN646074.1
B14	*Staphylococcus epidermidis*	89.7	89.7	100%	3e-15	98%	AE015929.1

Table S4. Staphylococci species isolated from chicken samples.

Isolates Code	Identification	Max Score	Total Score	Query Cover %	E-Value	Identity %	Accession
C1	*Staphylococcus epidermidis*	89.7	89.7	100%	3e-15	98%	CP000029.1
C2	*Staphylococcus aureus*	85.7	85.7	87%	5e-14	100%	JF170962.1
C3	*Staphylococcus aureus*	97.6	97.6	100%	1e-17	100%	FN646074.1
C4	*Staphylococcus aureus*	85.7	85.7	87%	5e-14	100%	JF170962.1
C5	*Staphylococcus epidermidis*	89.7	89.7	100%	3e-15	98%	FJ577582.1
C6	*Staphylococcus xylosus*	97.6	97.6	100%	1e-17	100%	KT351728.1
C7	*Staphylococcus epidermidis*	89.7	89.7	100%	3e-15	98%	AF269843.1
C8	*Staphylococcus aureus*	97.6	97.6	100%	1e-17	100%	FN646074.1
C9	*Staphylococcus aureus*	97.6	97.6	100%	1e-17	100%	FN646074.1
C10	*Staphylococcus aureus*	85.7	85.7	87%	5e-14	100%	JF170962.1

References

1. Clinical and Laboratory Standards Institute (CLSI). *Performance Standards for Antimicrobial Susceptibility Testing*; Twenty-Fourth International Supplements; Clinical and Laboratory Standards Institute: Wayne, PA, USA, 2014; p. 230.

© 2016 by the authors; licensee MDPI, Basel, Switzerland. This article is an open access article distributed under the terms and conditions of the Creative Commons by Attribution (CC-BY) license (http://creativecommons.org/licenses/by/4.0/).
